# The Effect of Low Carbohydrate Diets on Fertility Hormones and Outcomes in Overweight and Obese Women: A Systematic Review

**DOI:** 10.3390/nu9030204

**Published:** 2017-02-27

**Authors:** Melanie McGrice, Judi Porter

**Affiliations:** 1 Dietetics Department, Eastern Health, 5 Arnold Street, Box Hill VIC 3128, Australia; 2 Nutrition Plus Enterprises, 1004/1 Queens Road, Melbourne VIC 3004, Australia; 3 Allied Health Research Office, 5 Arnold Street, Box Hill VIC 3128, Australia; Judi.Porter@easternhealth.org.au; 4 Department of Nutrition, Dietetics and Food, Monash University, Level 1, 264 Ferntree Gully Road, Notting Hill VIC 3168, Australia

**Keywords:** low carbohydrate, ketogenic, polycystic ovarian syndrome, infertile, obese, overweight, systematic review

## Abstract

(1) Background: Medical interventions including assisted reproductive technologies have improved fertility outcomes for many sub-fertile couples. Increasing research interest has investigated the effect of low carbohydrate diets, with or without energy restriction. We aimed to systematically review the published literature to determine the extent to which low carbohydrate diets can affect fertility outcomes; (2) Methods: The review protocol was registered prospectively with Prospective Register for Systematic Reviews (registration number CRD42016042669) and followed Preferred Reporting Items For Systematic Reviews and Meta-Analyses guidelines. Infertile women were the population of interest, the intervention was low carbohydrate diets (less than 45% total energy from carbohydrates), compared to usual diet (with or without co-treatments). Four databases were searched from date of commencement until April 2016; a supplementary Google scholar search was also undertaken. Title and abstract, then full text review, were undertaken independently and in duplicate. Reference lists of included studies and relevant systematic reviews were checked to ensure that all relevant studies were identified for inclusion. Quality assessment was undertaken independently by both authors using the Quality Criteria Checklist for Primary Research. Outcome measures were improved fertility outcomes defined by an improvement in reproductive hormones, ovulation rates and/or pregnancy rates; (3) Results: Seven studies fulfilled the inclusion criteria and were included in the evidence synthesis. Interventions were diverse and included a combination of low carbohydrate diets with energy deficit or other co-treatments. Study quality was rated as positive for six studies, suggesting a low risk of bias, with one study rated as neutral. Of the six studies which reported changes in reproductive hormones, five reported significant improvements post intervention; (4) Conclusion: The findings of these studies suggest that low carbohydrate diets warrant further research to determine their effect. These randomised controlled trials should consider the effect of carbohydrates (with or without energy deficit) on hormonal and fertility outcomes.

## 1. Introduction

It has been suggested that approximately 7% of women of reproductive age have some form of sub-fertility [1,2]. Pre-conception weight is one of the major risk factors for fertility outcomes and it is well accepted that weight loss improves fertility in overweight and obese women [3,4,5]. Women with a body mass index (BMI) >30 kg/m^2^ are classed as being clinically obese [6] and have natural menstrual cycle disruptions at a rate of almost three times higher than women of a healthy weight [5]. A positive relationship also exists between pre-conception body mass index and the time needed to fall pregnant in women who are overweight and obese [5]. 

Carrying excess weight prior to conception can be an obstacle for obese women in getting pregnant, resulting in many women looking to healthcare professionals for assistance. Many overweight women who struggle with fertility have co-morbidities such as polycystic ovarian syndrome (PCOS) which poses additional challenges to fertility in itself due to disturbances in insulin resistance, sex-steroid metabolism and menstrual cycles. It has been estimated that 75% of infertile overweight or obese women have PCOS [7]. 

Although assisted reproductive technology such as in vitro fertilisation can provide an opportunity for these women and other sub-fertile couples to conceive, it is not a failsafe option. A retrospective cohort study conducted by Moragianni and colleagues showed that obese women have 68% lower odds of having a live birth following their first assisted reproductive therapy cycle compared to non-obese women [8]. Furthermore, obesity is related to a need for higher doses of assisted reproductive therapy medications, more frequent cycle cancellations and lower rates of efficacy at each stage of the in vitro fertilisation process [8]. It is well documented in the literature that weight loss can improve fertility and pregnancy outcomes, particularly involving in vitro fertilisation [9,10]. The benefits to this patient cohort include more regular menstrual cycles, better quality embryos available for transfer, less dosage requirement for medications and the need for fewer treatment cycles. Moreover, weight loss of as little as 5%–10% can be significant in improving hormonal imbalances, reducing rates of spontaneous abortions and miscarriages [1,2].

Pre-pregnancy weight loss has historically been centred on the traditional low fat, energy restricted diet plan. However the use of very low energy diets and low carbohydrate diets are increasingly being used to illicit more favourable weight loss and fertility outcomes. Low carbohydrate diets are those where less than 45% of macronutrients come from carbohydrates with or without an energy deficit [11]. A meta-analysis of overweight or obese adults with metabolic syndrome by Hu and colleagues found that low carbohydrate diets achieved comparable improvements in waist circumference, total-cholesterol, fasting glucose and serum insulin to low fat diets. Furthermore, low carbohydrate diets resulted in greater improvements in weight, high density lipoprotein cholesterol and triglycerides [11]. Concerns have been raised regarding the health effects of following low carbohydrate diets for long periods [12,13,14], however within particular clinical populations and for defined periods there may be benefits. 

This review aimed to systematically synthesise the published literature to determine the extent to which low carbohydrate diets can affect fertility outcomes. 

## 2. Materials and Methods 

This systematic review followed the Preferred Reporting Items For Systematic Reviews and Meta-Analyses (PRISMA) guidelines [15] and the protocol was prospectively registered with Prospective Register for Systematic Reviews (PROSPERO; registration number CRD42016042669). In a deviation from the published protocol, authors extended the comparator to enable a broader interpretation of the literature.

### 2.1. Eligibility Criteria

Criteria for study inclusion were developed using the Population—Intervention—Comparator—Outcomes format [16]. Overweight or obese infertile women were the population of interest, determined as a body mass index of ≥25 kg/m^2^ [6]. The intervention studied was low carbohydrate diets, defined as diets which comprised of less than 45% total energy obtained from carbohydrates, compared to usual diet (with or without co-treatments). Outcome measures were improved fertility outcomes defined by an improvement in reproductive hormones, ovulation rates and/or pregnancy rates. Original prospective research was eligible, but systematic reviews, commentaries and conference abstracts were excluded.

### 2.2. Search Strategy

The search terms were determined through exploration of key words used in the relevant literature and refined after consultation with a librarian with health science expertise. The subject headings and phrases were searched to ensure maximum retrievals. The search strategy focused on the population and intervention of interest. Outcomes were not defined within the search strategy, instead these were a focus of the title and abstract review.

Search terms used were as follows: “Diet, carbohydrate-restricted”, “low carbohydrate diet”, “low carbohydrate*”, “low carb“, “low carbs“, “carbohydrate restricted“, “ketogenic diet“, “ketogenic“, “ketosis“, “ketogenesis“, “very low energy diet“, “VLED“, “very low calorie diet“and “VLCD“. These terms were combined with “fertility“, “fertilisation“, “fertile“, “infertility“, “infertile”, ”fecundity”, ”fecundability”, “conceive”, “conception“, “pregnancy“ and “pregnant“. Animal studies were excluded. 

Study identification commenced by electronic searching from earliest available time until April, 2016, using CINAHL (from 1937), Ovid Medline (from 1946), Embase (from 1947) and the Cochrane Central Register of Controlled Trials (from 1991). No language exclusions were applied. A supplementary internet search was undertaken within Google Scholar to identify any studies that may have been missed through database searching. The search terms used were: “fertility and ketosis”, “fertility and VLED” and “fertility and low carbohydrate”. Reference lists of included studies and relevant systematic reviews were reviewed to ensure that the maximum number of studies were included. 

### 2.3. Study Selection

A title and abstract, then full text review of search retrievals was conducted by both authors against the inclusion criteria. Full texts were obtained of twenty papers. Once each reviewer completed the assessment any differences were discussed and resolved by consensus. At the completion of the full text review, the reference lists of included studies were reviewed to ensure that all eligible papers had been identified for the final library.

### 2.4. Data Extraction and Quality Assessment

A template was developed to extract data relating to participants, study duration, cause of infertility, study design, intervention, comparators and fertility outcome measures (impact upon reproductive hormones, ovulation rate and pregnancy rates). When an outcome measure was only reported by a single study (such as leptin [3]) data were not extracted. Data were extracted by one author and checked by a second author. 

Both authors independently rated study quality using the Quality Criteria Checklist for Primary Research [17]. This tool includes criteria that are associated with decreased bias and improved validity in primary research and is specific for studies in the field of nutrition and dietetics. Ratings of negative (weak quality; does not adequately address inclusion/exclusion, bias, generalisability, data collection and analysis), neutral (neither exceptionally strong, nor exceptionally weak quality) or positive (strong quality; adequately addresses inclusion/exclusion, bias, generalisability, data collection and analysis) were assigned, with consensus obtained through discussion. Where details were not provided or authors considered an “unsure” response for specific aspects within each validity question, a final response of “no” was made.

### 2.5. Analysis

Eligible studies were grouped according to the outcome measures and results were described narratively, with a greater emphasis placed on findings from studies achieving high-quality ratings. The mean and standard deviation or standard error of the mean were reported for each fertility outcome. Data were estimated from graphs where necessary. Due to the heterogeneity of study designs, interventions and outcomes, a meta-analysis was not undertaken.

## 3. Results

**Figure 1:**
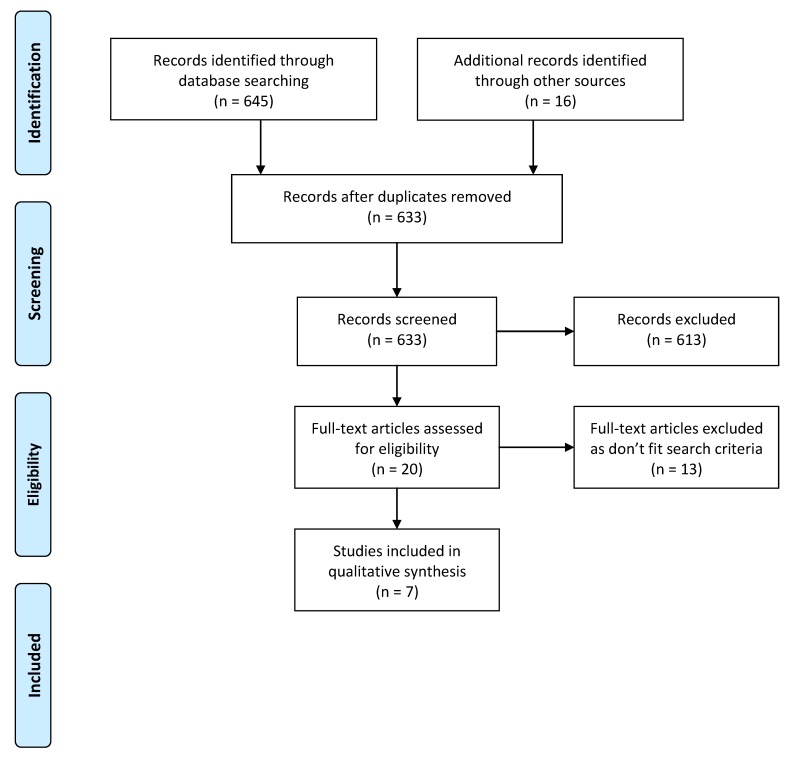
Study selection process.

Six hundred and forty-five papers were identified through database and internet searching and reference checking. With the removal of duplicates and animal studies, 62 studies were included for title and abstract review (Figure 1). No previous systematic reviews on this research question were located. 

Twenty studies were reviewed in full text, with seven studies identified that fulfilled the inclusion criteria for this review (Table 1). The included studies were small to moderate trials ranging from 11 to 96 participants. The researchers used a variety of study designs including two arm pre/post [18,19], matched control trial [20], randomised controlled trial [3,21] and randomised parallel studies [22,23]. All studies were conducted within the last fifteen years. The studies were undertaken by four groups of researchers from the USA, Italy or Australia, with intervention periods ranging from one month [21] to six months [18,22]. The participants in all studies, except Sim et al. trial had PCOS [3]. 

Included studies all utilised a diet of less than 45% of total energy obtained from carbohydrates. Approaches to dietary interventions varied considerably and included: ketogenic diets of less than 20 grams of carbohydrate per day with ad libitum energy intake [18], a very low energy diet with 34% carbohydrates [3], prescriptive energy restricted diets with 40% carbohydrates [20,21,23], and 45% carbohydrate diets with an energy deficit [19,22]. A variation was also seen in the control groups, where usual diet may have had restricted energy (to match the energy intake of the intervention group), or other co-treatment.

### 3.1. Study Quality

Table 2 shows that six studies (85%) were rated as positive, suggesting a low risk of bias [3,19,20,21,22,23]. One study was assessed as neutral quality as it was a two arm pre/post study instead of a randomised control trial and it was unclear whether or not subjects, clinicians and/or investigators were blinded [18]. The length of the intervention period in some studies may have limited hormone and fertility outcomes.

### 3.2. Outcomes

Outcome measures of the intervention groups were compared to the control groups (Table 3).

Six of the seven studies assessed changes in reproductive hormones [18,19,20,21,22,23] with all studies except Stamets and colleagues reporting significant improvements (*p* < 0.05). Mavropoulos (2005), Moran (2003), Palomba (2008) and Palomba (2010) all reported significant improvements in fasting insulin and testosterone, and although Thomson and colleagues reported an improvement in testosterone, their results were not significant [18,19,20,22]. Stamets and colleagues reported an improvement in insulin and testosterone compared to baseline data, but the improvement was not significant compared to their comparator [21]. Moran (2003), Palomba (2008), Palomba (2010) and Thomson (2008) all investigated sex hormone binding globule and free androgen index, and all noted significant improvements with increased production of sex hormone binding globule and a consequential lowering of the free androgen index (*p* < 0.05) [19,20,22,23]. Moran (2003), Palomba (2008), Palomba (2010) and Thomson (2008) also investigated fasting glucose, and although all studies reported improvements, only Thomson and colleagues results were statistically significant [20,22,23]. Stamets et al. 2004 investigated glucose using a glucose tolerance test but did not find a statistically significant improvement compared to the usual energy restricted diet [21]. Stamets and colleagues (2004) also reported follicle stimulating hormone and luteinizing hormone and again reported no statistically significant difference between their invention and control group findings [21]. Conversely, Moran (2003) and colleagues reported the luteinizing hormone/follicle stimulating hormone ratio and noted a significant improvement result in their intervention group [20]. 

Four of the seven studies reported menstrual cyclicity, frequency of menses and/or ovulation rates [19,20,22,23]. All of these studies illustrated a significant improvement in menstrual cyclicity and/or ovulation rates with a low carbohydrate diet. Palomba et al. demonstrated an improvement in mensus frequency and ovulation rates compared to the start of the intervention, but the results were not as significant as the usual diet plus structured exercise training [19]. The follow up study, found a significant improvement in ovulation rates (*p* = 0.020) between the control group who followed their usual diet plus clomiphene citrate, and the intervention group who followed a structured exercise program, clomiphene citrate and a 45% carbohydrate diet [22]. Moran and colleagues found a significant improvement in menstrual cyclicity with a 6000 kilojoule energy restricted diet [20]. Of the fourteen women following the low carbohydrate diet, four had an improvement in menstrual cyclicity, one amenorrheic subject had an improvement in ovulation and another amenhorrheic subject had a resumption of menses (42% improved menstrual cyclicity) [20]. Thomson and colleagues demonstrated a 21.4% improvement in menstrual cyclicity with a 5000–6000 kilojoule 40% carbohydrate diet [23].

Four studies reported pregnancy outcomes, with three of the four demonstrating improved pregnancy rates in the intervention group [3,18,19,20]. Of the five women who completed the intervention of less than 20 grams carbohydrates per day, two became pregnant (40%) [18]. A study by Moran and colleagues achieved two pregnancies with a 6000 kilojoule, 40% carbohydrate diet, but only one pregnancy in the control group with a 6000 kilojoule, 55% carbohydrate diet [20]. Palomba et al. achieved two pregnancies in twenty infertile women (10%) after commencing women on a 45% carbohydrate diet with an 800 kilocalorie (3300 kilojoule/day) deficit [19]. However, this was less than the control group who ate their usual diet, but commenced three structured exercise sessions per week, which resulted in seven pregnancies (35%) [19]. Sim and colleagues (2014) achieved thirteen pregnancies out of twenty-seven women in their intervention group (48%) with three of the thirteen being unassisted, which was statistically significant compared to a total of three assisted pregnancies (13%) in their control group [3].

## 4. Discussion

Effective, evidence-based strategies for optimising fertility are essential. This review aimed to identify and synthesise the evidence relating to the effect of low carbohydrate diets on fertility hormones and outcomes in overweight and obese women. There is convincing evidence that reducing carbohydrate load can reduce circulating insulin levels, improve hormonal imbalance and result in a resumption of ovulation to improve pregnancy rates. To this end, the findings of this review suggest that low carbohydrate diets may optimise fertility in some clinical groups, particularly for overweight and obese women with PCOS. At this stage there is no clear indication about whether low carbohydrate diets are as effective in overweight women without PCOS as only one study was found investigating this intervention with this patient group [3]. 

Findings of this systematic review support other literature on this topic. A prospective study of 18,555 women by Chavarro and colleagues also found that the quality of carbohydrate in the diet impacted the risk of ovulatory infertility with a 78% greater risk for women with higher carbohydrate consumption [9]. Numerous studies have shown that low carbohydrate diets not only elicit fast and significant weight loss but also reduce serum insulin, consequently improving insulin sensitivity [24,25]. The biochemical cascade which follows promotes a more favourable hormonal balance, with a reduction in free testosterone and increase in sex hormone binding globulin which can be associated with an improvement of menstrual function and fertility. The two-fold benefits of weight loss and improved hormonal balance also improve the clinical results for women with and without PCOS ultimately improving fertility [26]. However, it is important to note that energy restriction may be more salient than the macronutrient profile [27].

Furthermore, it is unclear how low in carbohydrates the diet should be or how long the diet should be maintained for optimal fertility outcomes. A small prospective study by Tsagareli et al. (2006) using meal replacements found that the six women who completed the study had less oocytes collected at the time of in vitro fertilisation after taking meal replacements than beforehand, even though they lost significant amounts of weight [28]. Unfortunately no pregnancies were achieved. As ketogenic diets, and very low energy diets in particular have been found to improve metabolic and hormonal variables in overweight and obese women, the study authors hypothesised that this effect may be a result of ketosis impacting upon oocyte quality [28]. Conversely, the study by Sim et al. using very low energy diets analysed in this review, found positive results on fertility [3]. A systematic review on the effect of weight loss on fertility noted that although using very low energy diets increased fertility outcomes, results were not as high as those seen in lifestyle interventions [5]. Kulak and Polotsky (2013) argue that there is scientific plausibility that a ketogenic diet should enhance fertility in certain populations [1]. However, one of the common criticisms about low carbohydrate diets is often that they result in weight regain [25]. A pattern of significant weight loss followed by a period of slight weight-regain is often utilised to optimise fertility in dairy cows [29]; a practice known as “flushing” which is used to improve the fecundity of farm animals. This pattern of a period of weight loss, followed by a period of weight regain was also found to demonstrate a positive impact on reproduction in women [20]. Consequently, it is recommended that more research be undertaken into the duration, timing and benefits of refeeding of women on low carbohydrate diets to best optimise fertility.

A particular strength of this review is that there were no time or language restrictions placed on the search strategy. Limitations include that there is no consensus in the literature to define “low carbohydrate”; the decision was made for a cut-off of 45% carbohydrate within this review. Furthermore, this review identified only one study for overweight women with non-PCOS related infertility, reinforcing the paucity of research in this area. Additional research into the impact of low carbohydrate diets on overweight women who don’t have PCOS would add weight to these findings. A further limitation of this study was the inconsistent reporting of exercise and/or behavioural interventions as co-treatments, so they have not been explicitly reported in this review. Randomised controlled trials investigating the optimum amount of carbohydrate (as a percentage of energy or maximum amount, with or without energy deficit) and timing of the intervention in relation to attempted pregnancy would also provide valuable progress in our understanding of this dietary approach.

## 5. Conclusions

This review found that reducing carbohydrate load can reduce circulating insulin levels, improve hormonal imbalance and resume ovulation to improve pregnancy rates compared to usual diet. However, there has been a lack of research on the benefit of low carbohydrate diets for non-PCOS related infertile women. In view of the increasing number of overweight women struggling to fall pregnant, there is need for further research in this area. 

## Figures and Tables

**Table 1:** Studies of low carbohydrate diets in overweight and obese women measuring fertility hormones and other reproductive outcomes.

Author, Year, Location	Participants; Study Duration	Cause of Infertility	Study Design	Intervention	Comparators	Fertility Outcomes (Pregnancy Rate/Other)
Mavropoulos et al. 2005, USA [18]	11 women with PCOS and BMI >27 from the community; 24 weeks	PCOS	Two arm pre/post study	<20 g carbohydrate/day, ad libitum MJ/day (<6% carbohydrate if consuming 5 MJ/day)	Own control on usual diet (pre-intervention)	Reproductive hormones; Pregnancy rates
Moran et al. 2003, Australia [20]	45 overweight women with PCOS; 12 weeks	PCOS	Matched control trial	High protein, low carbohydrate (6 MJ—40% carbohydrate, 30% protein, 30% fat)	Usual diet (55% carbohydrate, 15% protein, 30% fat) but limited to 6 MJ/day	Reproductive hormones; Ovulation rates
Palomba et al. 2008, Italy [19]	20 obese PCOS patients with anovulatory infertility; 24 weeks	PCOS	Two arm pre/post study	High protein, low carbohydrate diet (35% protein, 45% carbohydrate, 20% fat) with 3.3 MJ deficit	Usual diet and 3 physical activity sessions per week	Reproductive hormones; Ovulation rates; Pregnancy rates
Palomba et al. 2010, Italy [22]	96 overweight or obese Clomiphene citrate resistant women; 2 weeks intervention, 4 weeks clomid (±intervention)	PCOS	Three arm randomised parallel controlled trial	Structured exercise + 35% protein, 45% carbohydrate, 20% fat diet with 4.2 MJ/day deficit + clomiphene citrate	Usual diet followed by clomiphene citrate	Reproductive hormones; Ovulation rate
Sim et al. 2014, Australia [3]	49 obese women planning ART; 12 weeks intervention, 12 month follow up	Mixed	Randomised controlled trial	Very low energy diet (2.5 MJ/day, 34% carbohydrate) for 6/52 followed by 6/52 hypocaloric diet	Usual diet	Pregnancy rates
Stamets et al. 2004, USA [21]	35 obese women with PCOS; 1 month	PCOS	Randomised controlled trial	Diet (40% carbohydrate, 30% protein, 30% fat with 4.2 MJ/day energy deficit)	Usual diet (55% carbohydrate, 15% protein, 30% fat) with a 4.2 MJ/day energy deficit	Reproductive hormones
Thomson et al. 2008, Australia [23]	94 overweight and obese women; 20 weeks	PCOS	Randomised parallel study (only one arm, “Diet Only“ was included in this review)	Diet (5–6 MJ/day, 30% protein, 40% carbohydrate, 30% fat)	Own control on usual diet (pre-intervention)	Reproductive hormones; Ovulation rates

MJ/day = megajoules per day, PCOS = polycystic ovarian syndrome, BMI = body mass index, ART = assisted reproductive therapy.

**Table 2:** Quality assessment of included studies ^a^.

Author	Validity Rating ^b^										Overall Rating	Examples of Reasons for Downgrading
	1	2	3	4	5	6	7	8	9	10		
Mavropoulos et al. 2005, USA [18]	Y	Y	N/A	Y	N/A	Y	Y	Y	Y	N	Neutral	Statement of the role of funding source not included.
Moran et al. 2003, Australia [20]	Y	Y	Y	Y	N	Y	Y	Y	Y	N	Positive	Statement of the role of funding source not included.
Palomba et al. 2008, Italy [19]	Y	Y	Y	N	N	Y	Y	Y	Y	Y	Positive	Patients self-selected their intervention groups. Dropout rates were reported, but no further explanation was provided.
Palomba et al. 2010, Italy [22]	Y	Y	Y	Y	Y	Y	Y	Y	Y	Y	Positive	Source of participants (e.g., whether consecutive) was unclear.
Sim et al. 2014, Australia [3]	Y	Y	Y	Y	Y	Y	Y	Y	Y	N	Positive	Two groups didn’t match all aspects of demographics/anthropometry explained by the strict randomisation technique used. Statement of the role of funding source not included.
Stamets et al. 2004, USA [21]	Y	Y	Y	Y	N	Y	Y	Y	Y	Y	Positive	No reporting of blinding throughout the study protocol.
Thomson et al. 2008, Australia [23]	Y	Y	Y	Y	N	Y	Y	Y	Y	Y	Positive	Unclear whether the study dietitian was blinded.

Y = response of “yes” to the validity question; N = response of “no” to the validity question; N/A = not applicable. ^a^ Assessed using The Quality Criteria Checklist for Primary Research [17]; ^b^ Validity items: [1] research question stated; [2] subject selection free from bias; [3] comparable study groups; [4] method for withdrawals described; [5] blinding used; [6] interventions described; [7] outcomes stated, measurements valid and reliable; [8] appropriate statistical analysis; [9] appropriate conclusions, limitations described; [10] funding and sponsorship free from bias. Validity items 2, 3, 6, 7 must be satisfied for a positive quality rating.

**Table 3:** Fertility outcomes of included studies.

Study (Author, Year)	Intervention Group	Control Group	*p*-Value
Reproductive hormones			
Mavropoulos, 2005 [18]	Free testosterone ng/dL 1.7	Free testosterone ng/dL 2.19	0.04
	Luteinizing Hormone/Follicle Stimulating Hormone ratio 1.21	Luteinizing Hormone/Follicle Stimulating Hormone ratio 2.23	0.03
	Fasting serum insulin mcIU/mL 8.2	Fasting serum insulin mcIU/mL 23.5	0.002
Moran, 2003 [20]	Fasting glucose (mmol/L) 5.42 ± 0.13	Fasting glucose (mmol/L) 5.31 ± 0.17	NS
	Fasting insulin (mU/L) 16.6 ± 2.4	Fasting insulin (mU/L) 12.8 ± 2.0	<0.01
	Sex Hormone Binding Globule (nmol/L) 25 ± 2.5	Sex Hormone Binding Globule (nmol/L) 35 ± 5	0.027
	Testosterone (nmol/L) 1.45 ± 0.2	Testosterone (nmol/L) 1.3 ± 0.1	0.01
	Free Androgen Index (nmol/L) 7 ± 1.5	Free Androgen Index (nmol/L) 4.5 ± 1	0.004
Palomba, 2008 [19]	Follicle Stimulating Hormone (mIU/mL) 4.2 ± 13.2	Follicle Stimulating Hormone (mIU/mL) −1.2 ± 3.2	NS
	Testosterone (nmol/L) −28.7 ± 11.7	Testosterone (nmol/L) −33.4 ± 14.3	<0.05
	Sex Hormone Binding Globule (nmol/L) 41.9 ± 19.1	Sex Hormone Binding Globule (nmol/L) 82.5 ± 30.6	<0.05
	Free Androgen Index (%) −18.1 ± 9.7	Free Androgen Index (%) −27.2 ± 9.2	<0.05
	Fasting glucose (mmol/L) 1.2 ± 8.6	Fasting glucose (mmol/L) 0.4 ± 4.1	NS
	Fasting insulin (pmol/L) −13.1 ± 8.6	Fasting insulin (pmol/L) −23.4 ± 10.0	<0.05
Palomba, 2010 [22]	Follicle Stimulating Hormone (mIU/mL) 4.9 ± 3.1	Follicle Stimulating Hormone (mIU/mL) 4.2 ± 1.2	NS
	Testosterone (nmol/L) 2.2 ± 0.6	Testosterone (nmol/L) 2.51 ± 0.9	<0.05
	Sex Hormone Binding Globule (nmol/L) 25.3 ± 3.2	Sex Hormone Binding Globule (nmol/L) 17.4 ± 3.1	<0.05
	Free Androgen Index (%) 10.8 ± 3.5	Free Androgen Index (%) 11.6 ± 3.7	<0.05
	Fasting glucose (mmol/L) 4.0 ± 1.7	Fasting glucose (mmol/L) 4.0 ± 1.5	NS
	Fasting insulin (pmol/L) 15.8 ± 3.9	Fasting insulin (pmol/L) 17.9 ± 4.2	<0.05
Stamets, 2004 [21]	Testosterone (ng/dL) −9 ± 21	Testosterone (ng/dL) −9 ± 18	0.96
	Luteinizing hormone (mIU/mL) 7 ± 30	Luteinizing hormone (mIU/mL) 2 ± 11	0.59
	Follicle Stimulating Hormone (mIU/mL) −1 ± 5	Follicle Stimulating Hormone (mIU/mL) 2 ± 4	0.12
	Area under Curve Insulin 3 h Oral Glucose Tolerance Test −2912 ± 13,562	Area Under Curve Insulin 3 h Oral Glucose Tolerance Test −8734 ± 12,218	0.26
	Area Under Curve Glucose 3 h Oral Glucose Tolerance Test −87 ± 2803	Area Under Curve Glucose 3 h Oral Glucose Tolerance Test −93 ± 2049	0.99
Thomson, 2008 [23]	Glucose (mmol/L) 4.96 ± 0.6	Glucose (mmol/L)5.32 ± 0.49	<0.01
	Insulin (mU/L) 13.5 ± 9.9	Insulin (mU/L) 17.7 ± 8.2	<0.01
	Testosterone (nmol/L) 2.09 ± 0.98	Testosterone (nmol/L) 2.36 ± 0.71	NS
	Sex Hormone Binding Globule (nmol/L) 31.5 ± 17.5	Sex Hormone Binding Globule (nmol/L) 27.4 ± 15.9	NS
	Free Androgen Index (%) 8.4 ± 6.6	Free Androgen Index (%) 11.2 ± 5.5	<0.01
**Ovulation rates**			
Moran, 2003 [20]	Improved menstrual cyclicity 6/14 42%	Improved menstrual cyclicity 5/14 35%	NR
Palomba, 2008 [19]	Menses frequency (# observed menses/no expected cycles, %) 18/118 15.3%	Menses frequency (# observed menses/no expected cycles, %) 28/107 26.2%	0.043
	Ovulation rate (# ovulatory cycles/# observed cycles, %) 18/119 15.1%	Ovulation rate (# ovulatory cycles/# observed cycles, %) 28/113 24.8%	0.032
Palomba, 2010 [22]	Ovulation rate 12/32 37.5%	Ovulation rate 3/32 9.4%	0.020
Thomson, 2008 [23]	Improved menstrual cyclicity 3/14 21.4%	Improved menstrual cyclicity 0/14 0%	NR
**Pregnancy rates**			
Mavropoulos, 2005 [18]	Pregnancy 2/5 40%	Pregnancy 0/5 0%	NR
Moran, 2003 [20]	Pregnancy (# pregnancies/# patients, %) 2/14 14%	Pregnancy (# pregnancies/# patients, %) 1/14 7%	NR
Palomba, 2008 [19]	Pregnancy (# pregnancies/# patients, %) 2/20 10%	Pregnancy (# pregnancies/# patients, %) 7/20 35%	0.058
Sim, 2014 [3]	Natural conception 3/27 0.1%	Natural conception 0/22 0%	0.11
	Pregnancy rate 13/27 48.1%	Pregnancy rate 3/22 13.6%	0.007

NS = not significant; NR = not reported; data extracted by review authors where required from published graphs, # = number.
